# Pertussis Epidemiology in Greece and Emerging Risk Groups during the Vaccination Era (1980–2008)

**DOI:** 10.1155/2012/303846

**Published:** 2012-09-16

**Authors:** Maria Theodoridou, Georgia Dargenta, Maria Aptouramani, Panagiotis Papastergiou, Anna Katsiaflaka, Kalliopi Theodoridou, Christos Hadjichristodoulou

**Affiliations:** ^1^First Department of Pediatrics, “Agia Sofia” Children's Hospital, University of Athens, 11527 Athens, Greece; ^2^Department of Hygiene and Epidemiology, Faculty of Medicine, University of Thessaly, 22 Papakyriazi Street, 41222 Larissa, Greece

## Abstract

To study the epidemiology of pertussis in Greece and epidemiologic changes throughout a period of twenty-nine years, we conducted a retrospective analysis of available data of pertussis cases for the past twenty-nine years (1980–2008) and a prospective analysis of hospitalized pertussis cases from a children's hospital in Athens for eight years (2001–2008). From 1980 through 2008, the incidence of pertussis in Greece declined from 11.2 to 0.05 cases per 100,000. Epidemic cycles occurring every 3 to 5 years were observed. Since pertussis circulation cannot be fully controlled by present immunization programs, efforts should be made to vaccinate infants at the recommended age, early diagnose, treatment as well as contact tracing of pertussis cases. Control of pertussis in social susceptible populations is necessary. A national program with adolescent and adult booster could decrease the circulation of *B. pertussis*. Despite an overall decrease for pertussis cases, pertussis is still a present and future challenge of public health service in Greece.

## 1. Introduction

Pertussis remains one of the most frequent vaccine preventable diseases worldwide. Pertussis is still an important infectious disease with high morbidity and mortality worldwide, especially among infants in whom it is one of the leading causes of mortality. Although most often a persistent but relatively benign respiratory illness, pertussis can result in serious consequences, such as pneumonia, seizures, encephalopathy, and death, especially among infants. Despite high immunization rates in infants and children in many countries, pertussis remains endemic, with epidemics superimposed every 3–5 years [1–3]. Although the inception of childhood pertussis immunization programs has significantly reduced the occurrence of the disease in children, waning vaccine-induced immunity from the old vaccine permits the disease to affect adolescents and adults (after about 7 to 20 years from natural infection and 4 to 12 years from immunization), who in turn transmit the disease to nonimmunized or incompletely immunized infants who are more vulnerable to disease-related complications and higher mortality [4, 5]. Moreover, there is a clear lack of awareness regarding loss of immunity and occurrence of the disease in older patients.

In recent years, acellular pertussis vaccines have been incorporated into the immunization programs of many developed countries, gradually replacing whole cell vaccines. The whole-cell vaccine has been initiated in the Greek national vaccination schedule since 1951, whereas the acellular type since 1997. In Greece booster vaccines were firstly used by doctors of the private sector but were incorporated in Greek national vaccination schedule in 2008. According to the Greek national vaccination schedule, children are immunized compulsory at ages 2, 4, 6 months and with two booster doses at 15–18 months and at 4–6 years old with a diphtheria, tetanus, and pertussis (DTaP) vaccine [6]. DTaP is also part of four childhood combination vaccines that include other vaccines (e.g., IPV, Hib, HepB). In 1998 the vaccine coverage for pertussis (three doses) in Greece at two years of age was 82% in relation to 82–99% in other European countries [7]. According to a survey on vaccination coverage conducted by the National School of Public Health in 2006, the percentage of vaccinated children against pertussis reached almost 99% for the first three doses, 98% for the fourth, and 90% for the fifth. Moreover the estimated full DTP coverage (three doses) according to the World Health Organization for the period 2000–2008 was 99% for the Greek pediatric population [8, 9].

In the majority of countries where pertussis is a notifiable disease, a case-based national surveillance system is in place. The surveillance of pertussis in Greece is based on notification, which was made obligatory by law since 1950. However, differences in case definitions, methods of diagnosis, and reporting surveillance systems make direct intercountry comparisons difficult. Prolonged cough may be the only clinical feature in adolescents and adults, who may present for diagnosis late or not at all. Even when they present, pertussis clinicians regard it as childhood disease. The general consensus is that pertussis is under diagnosed and underreported [10]. Despite underreporting, an increased incidence of infant, adolescent, and adult pertussis has been observed worldwide [11–13]. This increase has been following the increase in incidence which was observed in 1980s in USA and in the 1990s in Canada and several European countries, despite the high childhood pertussis vaccination coverage and the use of the pertussis vaccine [11, 14–20]. 

Several studies have suggested that mothers, siblings, adolescents, grandparents, and healthcare workers are a significant source of infection to infants. Most adolescents acquire infection from schoolmates and friends, whereas for adults the main sources are children and work colleagues [5, 13, 21]. Teachers, healthcare workers, and childcare workers could be at greatest risk of being exposed to and transmitting the disease [22]. In addition to efforts to improve pertussis immunization rates in children, the expansion of vaccination to target to specific age groups should be considered [23]. In a recent publication the resurgence of pertussis was related with the emergence of strains producing increased amounts of pertussis toxin [24].

We describe the epidemiology of pertussis in Greece and epidemiologic changes throughout a period of twenty-nine years by using routinely collected national surveillance data and data from a tertiary children hospital. 

## 2. Materials and Methods 

In order to identify pertussis trends in Greece, epidemiological parameters of pertussis were collected from all available sources including notification data from the Ministry of Health and Social Solidarity, the Hellenic Centre of Disease Control and Prevention (KEELPNO) and hospitalization data from “Agia Sofia” a Children's Hospital in Athens.

### 2.1. Retrospective Study

We conducted a descriptive analysis of the available mandatory notification data for the period 1981–2008 from the Ministry of Health and Social Solidarity (1981–1997) and the Hellenic Centre of Disease Control and Prevention (1998–2008). The second source of information was hospitalization data for the period 1980–2008 from the Department of Infectious Diseases from the University Children's Hospital of “Agia Sofia” in Athens which is a major national pediatric referral centre, serving approximately 45% of the population of metropolitan Athens (urban area) and accepts almost 15,000 pediatric admissions annually.

The notification system for Pertussis in Greece relies on the clinicians from both private and public hospitals and primary health care settings to report the disease. For the period 1981–1997 only aggregated data were collected by Ministry of Health. From 1998 to 2004 the notification system was reformed, and case-based information was collected. The system was reformed for a second time during 2004 Olympic Games, and a specific form for pertussis reporting has been used thereafter, in which risk factors are included. 

### 2.2. Prospective Study

In order to collect information for the transmission of pertussis, we also conducted a prospective study on hospitalized pertussis cases in the Department of Infectious Diseases of the University Children's Hospital of “Agia Sofia” using data for the period of 2001–2008. We collected demographic data of the patients, vaccination status, symptoms and duration of illness, medication, laboratory results and complications. Clinical examination was performed to all patients that were admitted with the possible diagnosis of pertussis. Three blood tests and two nasopharyngeal aspirates were collected from each patient for laboratory confirmation. All blood samples were tested for leukocyte count, C-reactive protein, type of cells and serolog. The first nasopharyngeal sample was used for *Bordetella pertussis* DNA examination with PCR technique. The second nasopharyngeal was obtained with Dacron swabs in order to isolate *Bordetella pertussis* by culture, while the last aspirate was tested for RSV antigen.

### 2.3. Case Definition

Clinical case definition includes a cough illness lasting at least 2 weeks with one of the following: paroxysms of coughing, inspiratory “whoop,” or posttussive vomiting, without other apparent cause. Laboratory criteria for diagnosis include one of the following: demonstration of a specific *Bordetella pertussis* antibody response in absence of recent vaccination, detection of specific nucleic acid, and the isolation of Bordetella pertussis from clinical specimen.

The pertussis case definition used in Greece, according to the Hellenic Centre of Disease Control and Prevention [25], includes compatible clinical symptoms alone (possible case), clinical symptoms in combination with epidemiological link to laboratory confirmed case (probable case), or laboratory confirmation (confirmed case).

### 2.4. Data Analysis

Statistical analysis was performed by using Epi-Info (version 3.4.3-CDC-Atlanta) and SPSS (version 15.0-Chicago) software. Descriptive statistical analysis was conducted calculating percentages of Pertussis cases by sex and age group. Moreover, proportional distribution of Pertussis cases by age group both notified cases and hospitalized cases was calculated. 

## 3. Results

### 3.1. Retrospective Study

**Figure 1:**
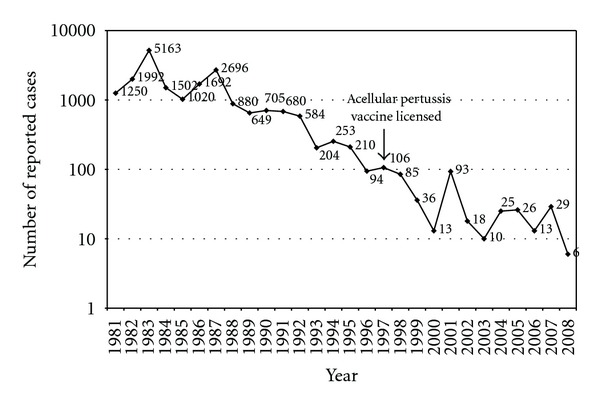
Semilogarithmic diagram of notified pertussis cases by year in Greece (1981–2008).

The annual incidence of pertussis in Greece delineated from the early 1980s (17.6 pertussis cases per 100,000) towards the 1990s at 2.1 pertussis cases per 100,000 and the early 2000s at 0.25 cases per 100,000, complying with the international epidemiological trends. However despite the overall decrease trend, small epidemic cycles are noted every three to four years (Figure 1).

**Figure 2:**
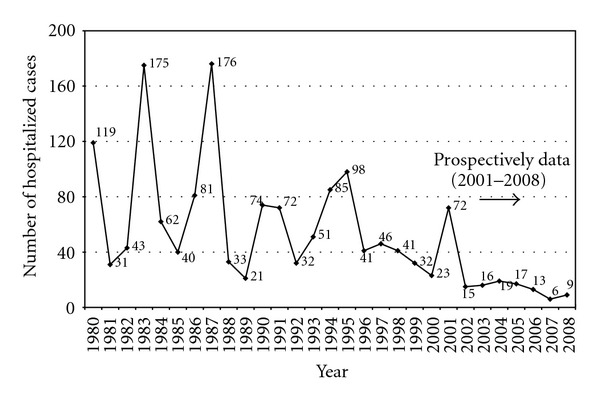
Yearly distribution of hospitalized pertussis cases in “Agia Sofia” Children's Hospital, Athens, Greece, 1980–2008.

Data of hospitalized pertussis cases from the Department of Infectious Diseases of “Agia Sofia” Children's Hospital were also available for the same time period of 1980–2008 (Figure 2). Yearly distribution of hospitalized pertussis cases for children followed the trends during the past thirty years in Greece.

### 3.2. Prospective Study

From January 2001 to December 2008, one hundred and sixty-seven children aged from 7 days until 14 years old were hospitalized for whooping cough in the Department of Infectious Diseases of “Agia Sofia” Children's Hospital. Among them 89 were males (53.3%) and 78 were females (46.7%). Eighty-four (50.3%) of the hospitalized patients were Greek, while 56 (33.5%) belonged to gypsy minority, and 27 (16.2%) were immigrants, while according to the census 2001 the population proportions of gypsy minority and immigrants are about 2.5% and 7%, respectively [26]. Eighty-two cases were confirmed by a PCR technique (49.1%). Serologic results were positive (Bordetella Pertussis-IgM or IgA positive) in 67 cases (40.1%), while both positive serologic and PCR results were found only in 16 cases (9.6%). In eighteen patients (10.8%) a PCR or a serological examination could not be performed; however an epidemiological link was identified (probable cases). 

### 3.3. Age Profile

**Figure 3:**
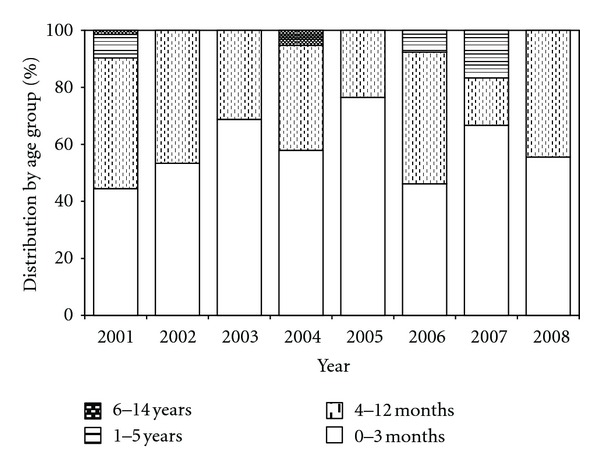
Age distribution of hospitalized Pertussis cases in the “Agia Sofia” Children's Hospital by year (2001–2008).

**Figure 4:**
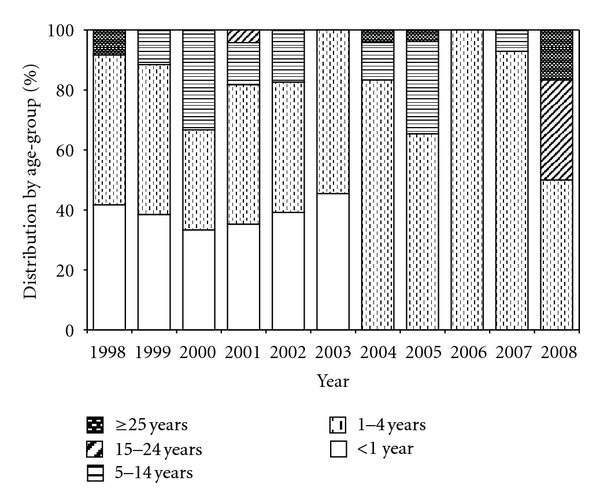
Age distribution of notified pertussis cases in Greece by year (1998–2008).

Among hospitalized for whooping cough in the “Agia Sofia” Children's Hospital ninety (53.9%) children were 0–3 months old, 67 (40.1%) were 4–12 months old, and 10 (6.0%) were from 13 months to 14 years old. Young infants aged 0–3 months old proved to be the most vulnerable population during the study period (Figure 3).  Figure 4 shows the age distribution of notified pertussis cases in Greece by year (1998–2008). 

### 3.4. Vaccination Status

Analysis of data of the health care and vaccination booklet of the hospitalized patients revealed that only 8 (4.82%) children were fully vaccinated according to the basic vaccination schedule against pertussis receiving three or more doses of DTaP (three patients aged 4–12 years old and five patients older than 12 months old). In total 158 (94.6%) hospitalized children were unvaccinated and insufficiently vaccinated against pertussis (having received DTP1 or DTP1 + DTP2), specifically: 90 out of 158 (57.0%) children aged 0–3 months old, 59 (37.3%) children aged 4–12 months old, and 9 (5.7%) children aged more than one year old. In total, ninety-nine (62.7%) of hospitalized children were insufficiently vaccinated because they were too young to have received three doses needed for protective immunity. 

### 3.5. Complications

Most infants and children suffered from ordinary respiratory complications: 52 (31.1%) presented with serious apnea episodes, 17 (10.2%) presented with acute bronchiolitis, 12 (7.2%) patients suffered from atelectasis, three patients suffered from pneumonia (1.8%), one with emphysema, and another one with pneumothorax. Five (3.0%) patients were admitted in the Intensive Care Unit, but successfully recovered. No encephalopathy case and no deaths were recorded. The mean duration of hospital stay was 9.8 days for infants 0–3 months old, 7.5 days for infants 4–12 months old, and 5.3 days for children older than the first year of life.

## 4. Discussion 

The past thirty years data on pertussis morbidity in Greece demonstrates a decrease in endemic. Small epidemic cycles were noted every three to four years, an epidemiological characteristic which has been extensively described in the literature [1]. The increase in vaccination coverage at high levels during the study period [8], the implementation of surveillance and a series of occasional vaccination programmes focused on groups such member of the Roma community and other ethnic minorities during the last decades were important factors for the observed decreased morbidity of pertussis. 

We observe that there are discrepancies between hospital admissions and notifications for children less than one year of age for the period 2004–2008. These differences are due to the underreporting of the notification system in Greece. However, we did not observe a significant age distribution shift among cases admitted to the children's hospital from 2001 to 2008. Moreover, serious complications (encephalopathy or death) were not recorded in our study. We have no indication that cases of encephalopathy or death have been missed, since both of them are serious conditions and should be hospitalized and properly laboratory investigated. Literature [13, 27] suggests that mortality rate is higher in neonates and very young infants; however this was not observed in our study. 

The rate of increase of immigration into Greece since 1988 has been phenomenal, multiplying the number of immigrants fivefold. The legalisation programmes of 1997 and 2001 made an impact on the illegality of migrants, but of a transitory nature and leaving a large minority in illegal status. The greatest cluster of immigrants is in the Municipality of Athens counting for almost 17% of population; Thessaloniki has the second largest cluster, but reaching only 7% of local population [26]. 

After the introduction of vaccination, age-specific incidence was the highest in infants followed by preschool children. It seems that an age distribution change of notified pertussis cases in Greece since the 2000s has to lead to the emergence of groups at higher risk of the diseases in such young children, adolescents, and adults. A shift of the age distribution of reported cases to adolescence and adults has been reported furthermore in other developed countries [28–30]. Studies in other countries have provided evidence for circulating *B. pertussis* strains with increased virulence. An antigenic divergence occurred between vaccine strains and clinical isolates [24, 31] which may have resulted in reduced vaccine-induced immunity. Especially surface proteins of *B. pertussis* (pertussis toxin, pertactin, fimbriae) which confer protective immunity to the bacterium play a key role. However a molecular epidemiology typing study of *B. pertussis *isolates has not been conducted in Greece. Such study could furthermore give valuable epidemiological information. 

Every effort should be made to vaccinate infants at the recommended age and to diagnose and treat pertussis cases in children and adults in contact with infants. Despite the dramatic decrease of pertussis incidence, data from both sources indicated that we have an increase of the number of cases (outbreaks) every 3-4 years. This finding supports that *Bordetella pertussis* circulation cannot be controlled by present immunization programs. A program with adolescent and adult boosters will decrease the circulation of *B. pertussis* in these age groups and could possibly lead to the elimination or at least procuring a nonproliferation of the organism from the population. The recently implemented dTap immunization program for adolescents is expected to stop the increasing incidence in this age group. The Greek National Committee on Immunization recommends that all Greek adults should receive a single dose of dTap vaccine instead of a tetanus-diphtheria booster. 

Diagnosis of pertussis is a major barrier to the management of the outbreaks. A delay in diagnosis of pertussis in adults and adolescents increases the possibility of infection in toddlers. Symptoms frequently disorientate clinicians to other upper respiratory diseases. Considering the nature of the disease and the insufficiently registered epidemiological information regarding pertussis, it is obvious that the implication of a more reliable surveillance system is also required (e.g., laboratory reporting system or dedicated surveillance network [30], as well).

Prevention is possible through avoidance of exposure, chemoprophylaxis, and vaccination. Several strategies are considered for reducing the pertussis burden internationally. Specifically for Greece we propose certain Health Preventive methods such as reinforcement of current infant and toddler immunization strategies; effort should be made to diagnose in time and treat pertussis in children and adults in close contact with infants, selective immunization of mothers and close family contacts of newborns, national vaccination strategy for the immunization of social susceptible populations (Roma and especially immigrants), and adult immunization by the new dTap vaccine. Contact tracing followed by treatment and isolation (quarantine) could be an additional key control measure in the battle against infectious diseases and can be routinely implemented in cases with *B. pertussis* [32–34]. Recently publication suggests that contact structure is the pivotal element for understanding the epidemiology of pertussis [35]. 

Despite the increase in vaccination coverage for pertussis at high levels in Greece during the study period, challenges still remain. A recent study estimated the percentage of all rates of booster dose of tetanus/diphtheria/pertussis at 39.6% [36]. Implementation of vaccination programs, chemoprophylaxis, and contract tracing remains problematic in susceptible populations such as Roma and immigrants due to social and cultural circumstances. The presence of clusters of immigrants and especially the great cluster of immigrants in Athens with low accessibility to health care facilities could possible result in a future public health threat. The number of illegal immigrants is continuously increasing. Recently changes in the Greek immigration policy could have negative impact on accessibility, especially for illegal immigrants, to healthcare facilities because of the fear of repatriation. An implementation of an obligatory adult immunization by the new dTap vaccine is difficult and widely enforced. Moreover the recently debt crisis in Greece will possibly have a negative impact on the process of many running or future planed public health programs. Due to limited resources, evaluation and an audit of the proposed measures are therefore needed in order to prioritize the measure and achieve the optimum impact with fewer resources in pertussis control in Greece. 

## 5. Conclusions

The annual incidence of pertussis in Greece was delineated in the last decades. Pertussis circulation cannot be controlled by present immunization programs. Control of pertussis in social susceptible populations (Roma and emigrants), universal recommendation for an additional adolescent booster, vaccination of persons with close contact to infants as well as contact tracing could further reduce morbidity. Despite an overall decrease for pertussis cases, pertussis is still a present and future challenge of public health service in Greece.

## Abbreviations

*R*^2^: Coefficient of determination
DTaP: Diphtheria, tetanus, and pertussis/acellular vaccine
dTaP: Diphtheria, tetanus, and pertussis/acellular vaccine for adolescents
IPV: Inactivated poliovirus vaccine
Hib: Haemophilus influenzae type b vaccine
HepB: Hepatitis B vaccine
DTP: Diphtheria, tetanus and pertussis vaccine
PCR: Polymerase chain reaction
RSV: Respiratory syncytial virus.
